# Cardiac myxoedema due to severe hypothyroidism mimicking myopericarditis: a case report

**DOI:** 10.1093/ehjcr/ytag587

**Published:** 2026-08-25

**Authors:** Elena Gualini, Patrizia Pedrotti, Paolo Dalino Ciaramella, Giulia Maida, Lucia Occhi, Piero Gentile, Cristina Giannattasio

**Affiliations:** Cardiology Department, De Gasperis Cardio Center, ASST Grande Ospedale Metropolitano Niguarda, Piazza Ospedale Maggiore 3, 20162 Milan, Italy; Cardiology Department, De Gasperis Cardio Center, ASST Grande Ospedale Metropolitano Niguarda, Piazza Ospedale Maggiore 3, 20162 Milan, Italy; Endocrinology Department, ASST Grande Ospedale Metropolitano Niguarda, Piazza Ospedale Maggiore 3, 20162 Milan, Italy; Endocrinology, Diabetology and Medical Andrology Unit, IRCCS Humanitas Research Hospital, Via Manzoni 56, 20089 Rozzano, Milan, Italy; Cardiology Department, De Gasperis Cardio Center, ASST Grande Ospedale Metropolitano Niguarda, Piazza Ospedale Maggiore 3, 20162 Milan, Italy; Cardiology Department, De Gasperis Cardio Center, ASST Grande Ospedale Metropolitano Niguarda, Piazza Ospedale Maggiore 3, 20162 Milan, Italy; Cardiology Department, De Gasperis Cardio Center, ASST Grande Ospedale Metropolitano Niguarda, Piazza Ospedale Maggiore 3, 20162 Milan, Italy; School of Medicine and Surgery, University of Milano-Bicocca, Piazza dell'ateneo nuovo 1, 20126 Milan, Italy

**Keywords:** Hypothyroidism, Cardiac myxoedema, Myocardial oedema, Cardiac magnetic resonance, Myopericarditis, Case report

## Abstract

**Background:**

Severe hypothyroidism may present with cardiac manifestations, including pericardial effusion and myocardial oedema, which can mimic inflammatory myocardial and pericardial diseases. Recognition of this potentially reversible condition is crucial in the differential diagnosis of patients presenting with suspected myopericarditis.

**Case summary:**

A 42-year-old woman presented with recurrent chest pain initially suspected as relapsing myopericarditis following a recent SARS-CoV-2 infection. The electrocardiogram showed sinus rhythm with diffuse low-voltage QRS complexes. Transthoracic echocardiography demonstrated preserved biventricular systolic function and a circumferential pericardial effusion measuring up to 8 mm, without echocardiographic signs of cardiac tamponade. Laboratory tests revealed a markedly elevated creatine phosphokinase (>3000 U/L), mild troponin elevation, and severe hypothyroidism (thyroid-stimulating hormone: 538 µU/mL) with positive thyroid antibodies. Cardiac magnetic resonance demonstrated diffuse biventricular myocardial oedema and mild-to-moderate pericardial effusion without late gadolinium enhancement. In the context of severe hypothyroidism, these findings were considered consistent with cardiac myxoedema. Thyroid hormone replacement resulted in rapid clinical and echocardiographic improvement. The overall clinical, biochemical, and imaging findings supported severe hypothyroidism as the underlying cause.

**Discussion:**

This case highlights severe hypothyroidism as a reversible cause of myocardial oedema that may mimic inflammatory myopericarditis and should be considered in the differential diagnosis of patients presenting with myocardial oedema and pericardial effusion.

## Graphical Abstract

**Graphical Abstract:**
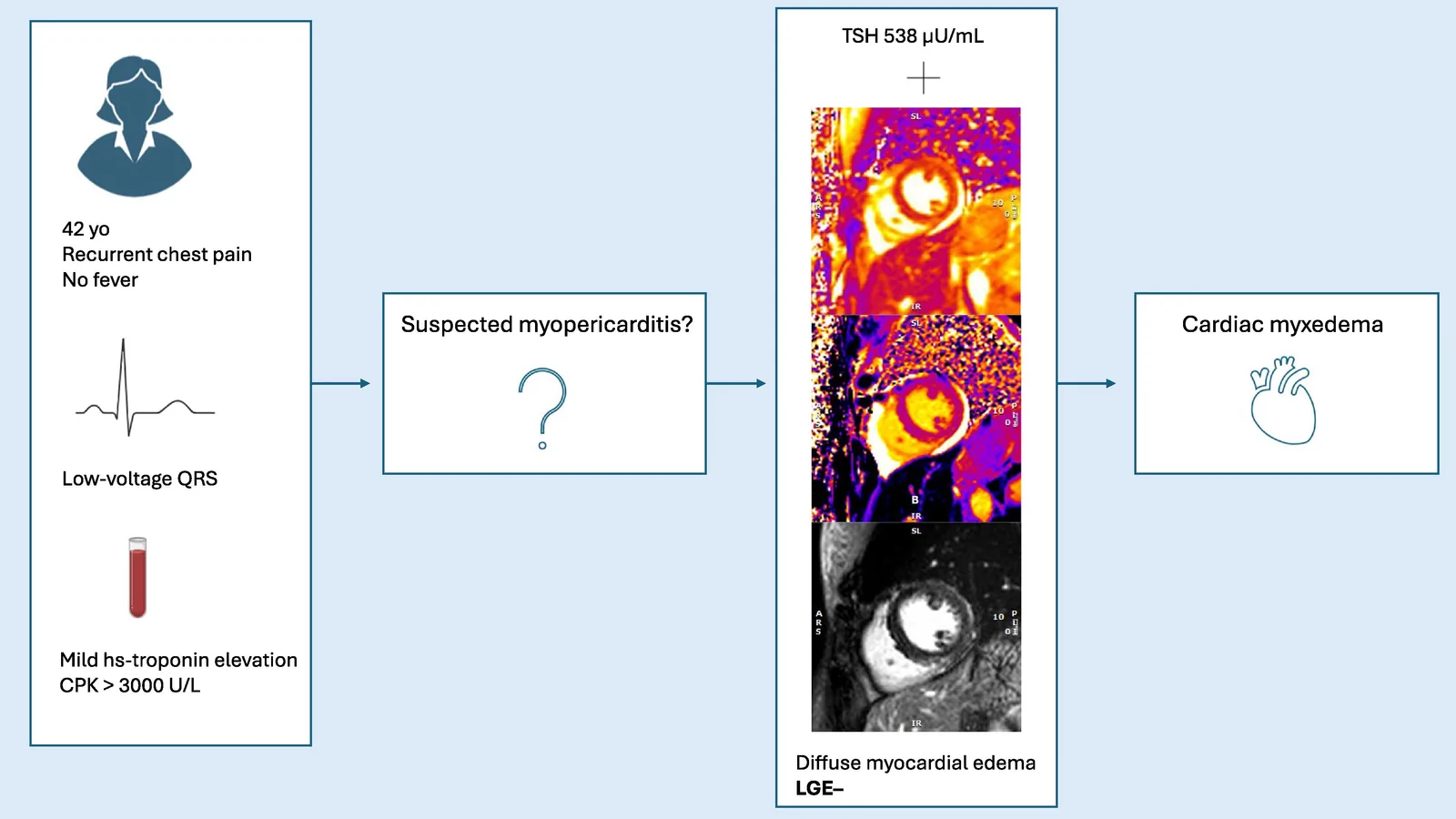

Learning points

- Severe hypothyroidism may cause myocardial and pericardial oedema that can mimic inflammatory myopericarditis.
- Cardiac magnetic resonance imaging showing diffuse myocardial oedema without late gadolinium enhancement may suggest a non-inflammatory aetiology.
- Thyroid function testing should be considered in patients presenting with unexplained myocardial oedema or suspected myopericarditis.

## Introduction

The thyroid and cardiovascular systems are closely interconnected, and complications may arise in both subclinical and overt thyroid disease. ^1,2^ In hypothyroidism, patients can present with increased vascular resistance, low cardiac output, reduced stroke volume, diminished intravascular volume, prolonged circulation time, and extended diastolic relaxation.^3^

Although uncommon, severe hypothyroidism can present with myocardial and pericardial oedema, potentially mimicking inflammatory cardiac conditions such as myopericarditis and leading to diagnostic uncertainty. Cardiac magnetic resonance (CMR) plays a key role in myocardial tissue characterization and in the differential diagnosis of myocardial oedema.

We describe a case of severe hypothyroidism presenting with diffuse myocardial oedema on CMR without late gadolinium enhancement, initially suspected as relapsing myopericarditis, highlighting an important diagnostic pitfall and the role of thyroid function testing in unexplained cases.^4,5^

## Case presentation

A 42-year-old woman presented to the emergency department (ED) in August 2022 for chest pain not related to effort. She had had three full-term pregnancies and had been amenorrheic for the past 2 years, but she had not sought medical attention. During her last pregnancy, she developed hypothyroidism requiring levothyroxine, which was discontinued after delivery.

In June 2022, she had been admitted to hospital for suspected COVID-19-related pneumonia and pericarditis, presenting with chest pain and cough (troponin T level 14 ng/L). She was treated with ibuprofen and colchicine and discharged after a few days.

**Figure 1:**
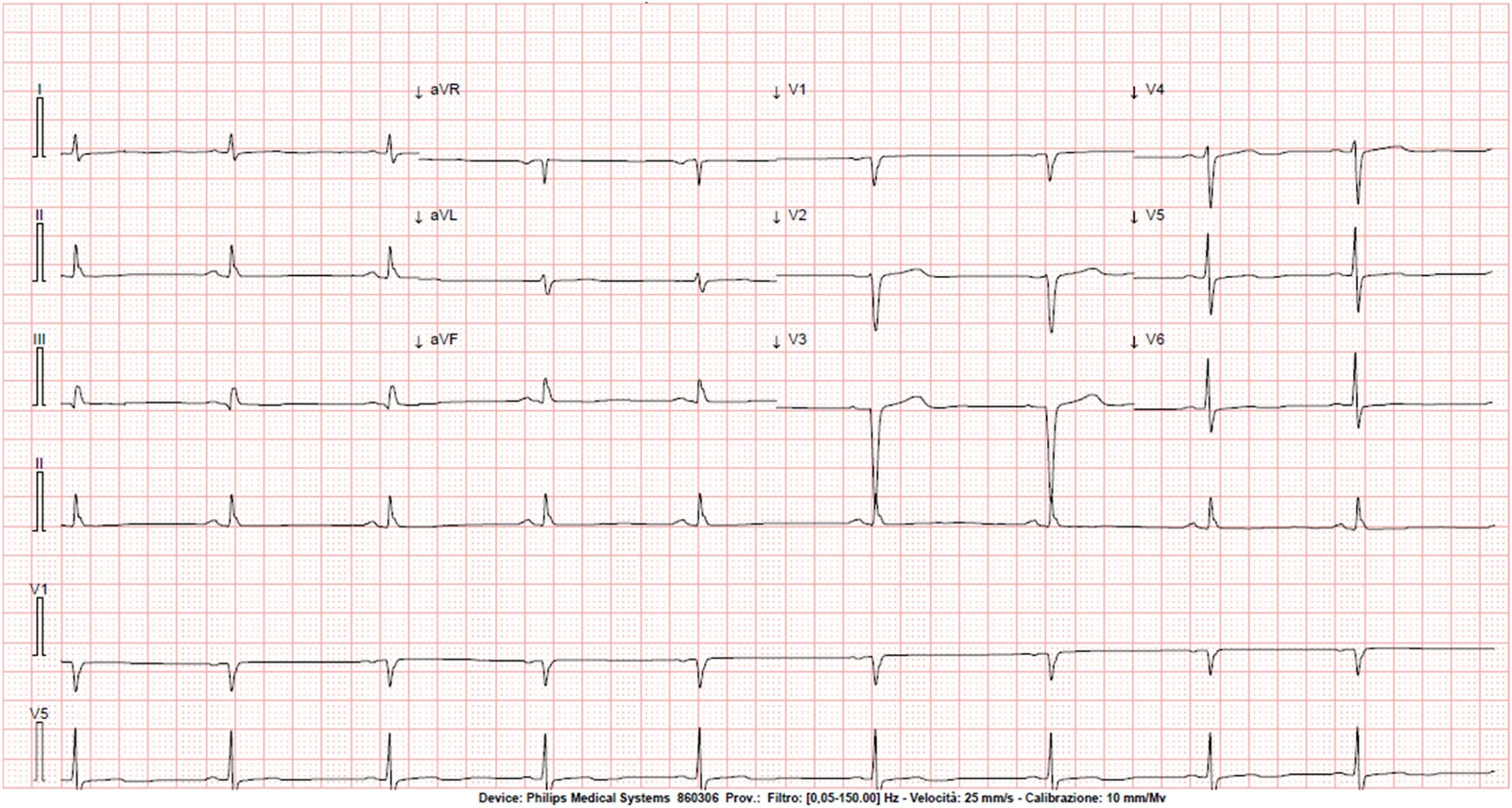
12-lead electrocardiogram showing sinus rhythm, low QRS voltage in peripheral leads, pseudonecrosis pattern in V1–V3, and diffuse repolarization abnormalities.

In August 2022, she returned to the ED with worsening chest pain and myalgia following tapering of her medications. On examination, she was afebrile and had facial swelling and a hoarse voice. A 12-lead electrocardiogram showed sinus rhythm at a normal rate, low voltage on peripheral leads, pseudonecrosis pattern in V1–V3, and mild diffuse repolarization abnormalities (*Figure 1*).

Transthoracic echocardiography demonstrated preserved biventricular systolic function and a circumferential pericardial effusion measuring up to 8 mm, without echocardiographic signs of cardiac tamponade. Laboratory testing showed no elevation of inflammatory markers, a mild increase in high-sensitivity troponin T (peak 19.1 ng/L; normal range 0–14), and markedly elevated creatine phosphokinase (CPK) (peak 3155 U/L; 464 at discharge; normal range 30–150). She was admitted with a suspected diagnosis of relapsing myopericarditis. Thyroid tests demonstrated severe hypothyroidism with markedly elevated thyroid-stimulating hormone (TSH) (peak 538 µU/mL; 322 at discharge; normal range 0.27–4.2) and positive thyroid autoantibodies, consistent with Hashimoto’s thyroiditis.

**Figure 2:**
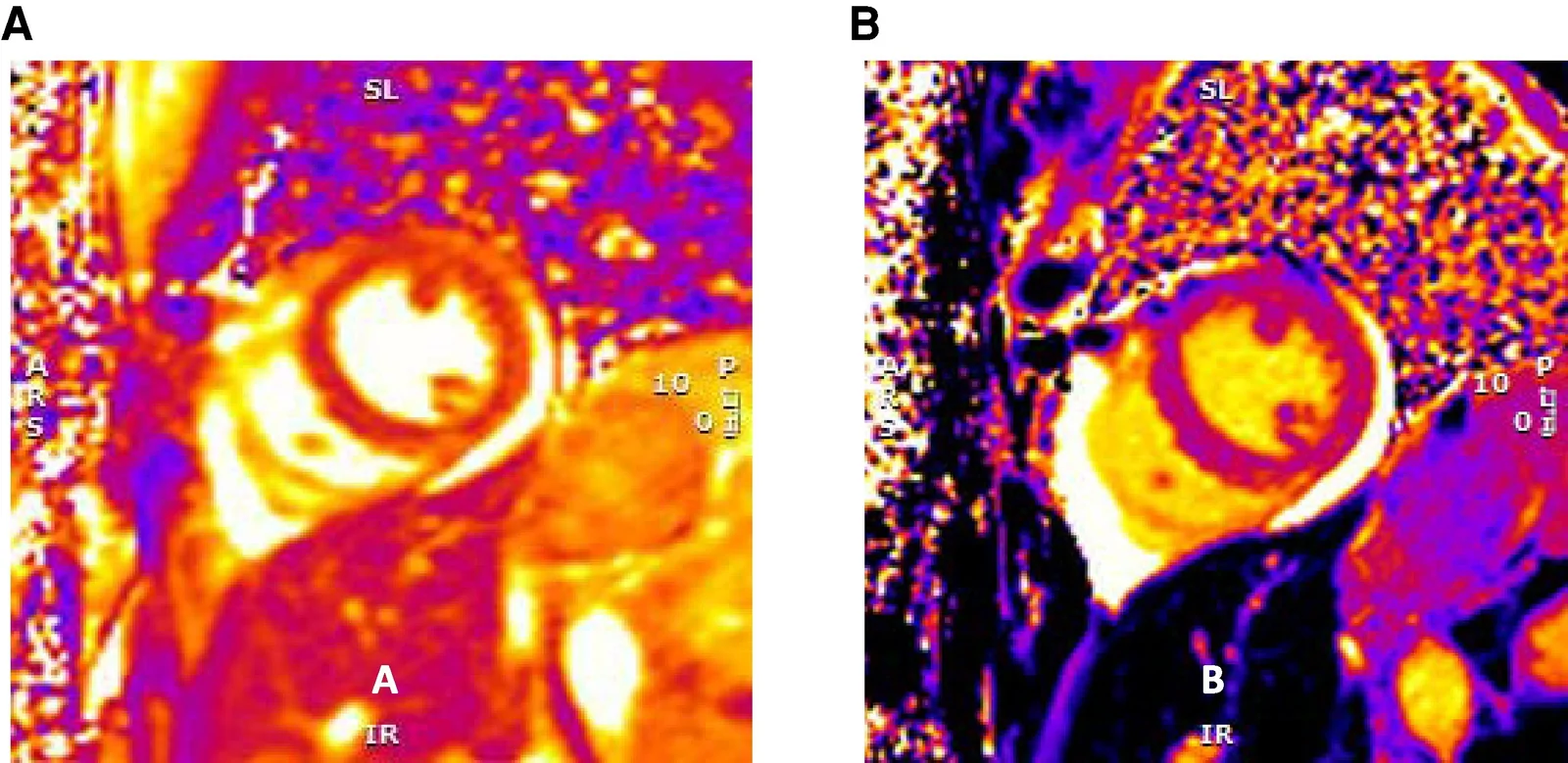
Cardiac magnetic resonance parametric mapping. (*A*) T2 mapping (TrueFISP sequence) on a mid-ventricular short-axis slice demonstrating diffuse elevation of myocardial T2 values (global circumferential value across three short-axis slices: 63 ms; laboratory reference value 45 ± 2 ms), consistent with diffuse myocardial oedema. (*B*) Native T1 mapping (MOLLI sequence) on a mid-ventricular short-axis slice showing diffuse elevation of myocardial T1 values (global circumferential value across three short-axis slices: 1159 ms; laboratory reference range 983 ± 30 ms), supporting diffuse myocardial involvement. TrueFISP, true fast imaging with steady-state precession; MOLLI, modified look-locker inversion recovery.

**Figure 3:**
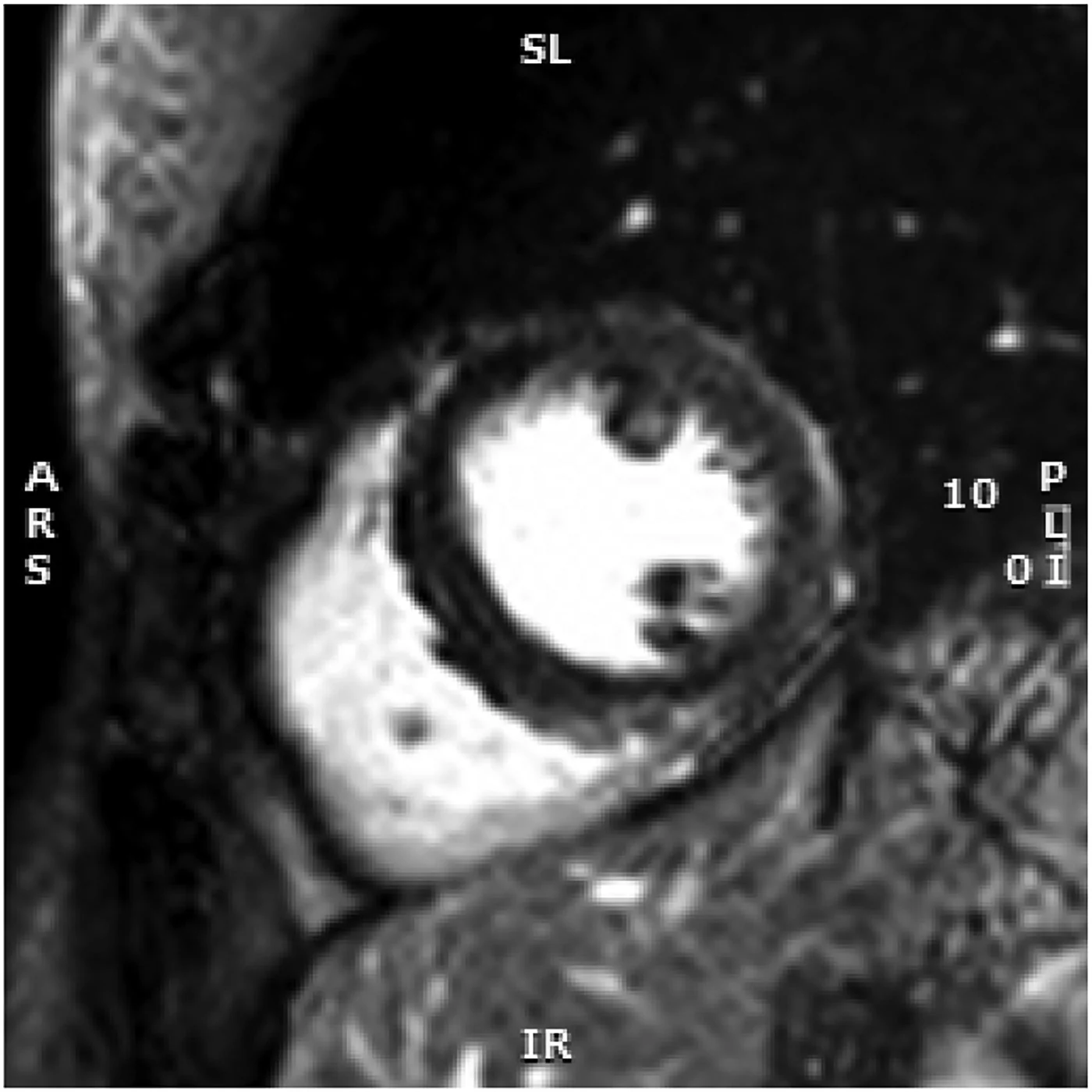
Post-contrast cardiac magnetic resonance image on a mid-ventricular short-axis view demonstrating the absence of late gadolinium enhancement.

CMR showed diffuse biventricular myocardial oedema: T2-weighted short tau inversion recovery sequences showed increased signal intensity with a myocardial-to-skeletal muscle signal ratio >2; global T2 mapping with true fast imaging with steady-state precession sequence elevated at 63 ms (laboratory range 45 ± 2 ms); global native T1 mapping with modified look-locker inversion recovery sequence elevated at 1159 ms (laboratory range 983 ± 30 ms); extracellular volume elevated at 35% (laboratory range 25 ± 3%). Biventricular function was normal, and there was mild to moderate pericardial effusion (*Figures 2* and *3*), without pericardial oedema or late enhancement. There was no myocardial late enhancement on post-contrast images.

**Figure 4:**
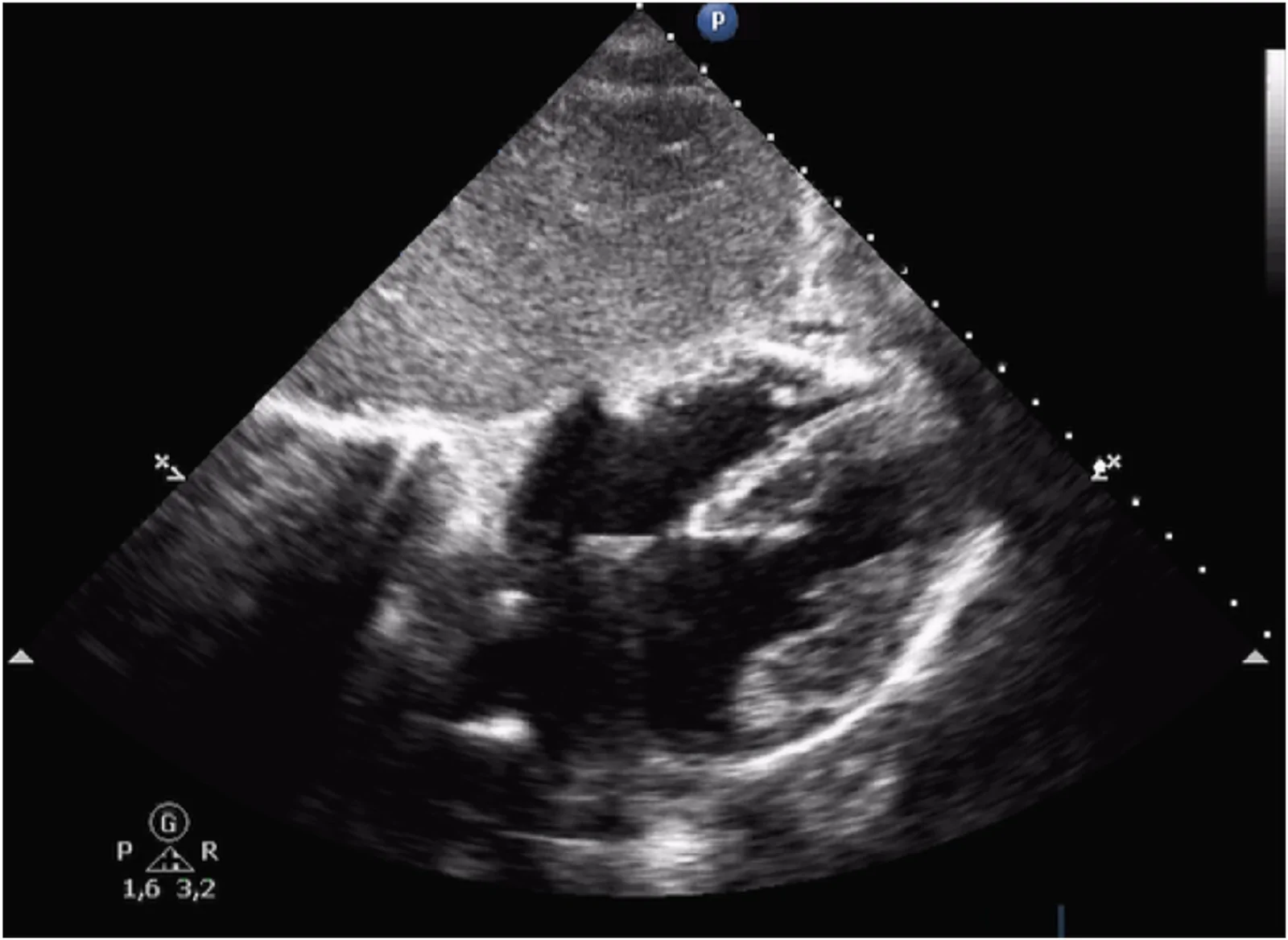
Follow-up transthoracic echocardiography obtained from a subcostal view 8 days after initiation of thyroid hormone replacement therapy, demonstrating marked reduction of the circumferential pericardial effusion from 8 mm to approximately 2 mm.

Treatment with low-dose levothyroxine and short-term liothyronine was initiated. Liothyronine was discontinued after a few days when FT3 normalized. Because of the severity of hypothyroidism, adrenal function was assessed before initiation of thyroid hormone replacement therapy and showed normal cortisol and adrenocorticotropic hormone (ACTH) levels (*Table 1*). At 8-day follow-up, echocardiography showed a reduction of the effusion to 2 mm (*Figure 4*).

**Table 1:** Laboratory findings at admission, discharge, and 2-month follow-up

	August 22nd	September 14th	November 15th
**Hb (12–16 g/dL)**	11.3	11.0	12.0
**WBC (**×**10^9^/L)**	7.00	7.48	4.3
**PLT (140–440** × **10^9^/L)**	251	280	291
**hs-cTn (0–14 ng/L)**	19.1	10.3	7
**CK-MB (0–5** µ**g/L)**	26.7	5.7	
**CPK (30–150 U/L)**	**3155**	**456**	**148**
**Creatinine (0.51–0.95 mg/dL)**	1.15	1.02	0.7
**CRP (mg/dL)**	0	0	0
**TSH (0.27–4.2 µU/mL)**	**538**	**322**	**2.9**
**FT3 (1.8–4.5 pg/mL)**	1.3	1.8	
**FT4 (9.2–17 pg/mL)**	0.7	5.6	
**Cortisol (62.4–180** µ**g/dL)**	75	97.7	
**ACTH (7.2–63.3 pg/mL)**	34.6		
**Anti-thyroglobulin (0–115 UI/mL)**	174		
**Anti-thyroperoxidase (0–34 UI/mL)**	84		
**Anti-TSH receptor (<1.2 UI/L)**	1.4		

The bold values is to emphasize the quick changes after therapy was started.

ACTH, adrenocorticotropic hormone; CK-MB, creatine kinase myocardial band; CPK, creatine phosphokinase; CRP, C-reactive protein; FT3, free triiodothyronine; FT4, free thyroxine; hs-cTnT, high-sensitivity cardiac troponin T; PLT, platelet count; TSH, thyroid-stimulating hormone; WBC, white blood cell count.

At a November 2022 outpatient review, TSH was within the normal range (2.9 µU/mL) with corresponding normalization of CPK and hs-troponin levels. The patient reported marked clinical improvement and resumption of menses over the past 2 months (*Table 1*). A follow-up electrocardiogram was not available; therefore, the evolution of the low-voltage QRS complexes and pseudonecrosis pattern could not be assessed. A follow-up CMR examination had been scheduled but was not performed because the patient declined further imaging.

## Discussion

Severe hypothyroidism may present with a wide spectrum of cardiovascular manifestations, ranging from subtle haemodynamic changes to overt cardiac involvement. In the present case, the main diagnostic challenge was the differentiation between inflammatory myopericarditis and myocardial oedema related to severe hypothyroidism.

TSH, secreted by the pituitary gland, stimulates the thyroid to produce triiodothyronine (T3, 15%) and predominantly thyroxine (T4, 85%). T4 is converted to T3 via 5-monodeiodination in skeletal muscle, liver, and kidney. These hormones regulate metabolism and exert effects across multiple organ systems.^6^

The impact of thyroid hormones on cardiac myocytes is mainly mediated by T3, as these cells lack significant intracellular deiodinase activity; T3 is transported into the cell and regulates the expression of key structural and regulatory genes.

In hypothyroidism, reduced thyroid hormone activity leads to decreased myocardial contractility, increased systemic vascular resistance, impaired diastolic relaxation, and reduced cardiac output.^1^

In addition to these haemodynamic changes, severe hypothyroidism may cause interstitial accumulation of mucopolysaccharides and collagen causing swelling of the myocardial fibres and expansion of the interstitial space.^7^ When this process involves the myocardium and pericardium, it may manifest as cardiac myxoedema, characterized by myocardial and pericardial fluid accumulation.

While cardiac tamponade is rare, small-to-moderate pericardial effusions occur in up to 30% of patients with overt hypothyroidism. Importantly, it typically resolves after thyroid hormone replacement therapy, reflecting the reversible nature of the underlying pathophysiological process.^8^

In patients with severe hypothyroidism, early assessment of adrenal function (serum cortisol and ACTH) is essential to exclude concomitant adrenal insufficiency and to guide the possible use of adjunctive glucocorticoid therapy.^9^

Failure to diagnose this endocrine disorder may result in delayed initiation of specific therapy, with consequent failure to recognize and treat potentially serious but reversible cardiovascular manifestations. Therefore, it would be reasonable to check the thyroid status of all patients presenting with apparent myopericarditis or heart failure, in the absence of another clearly identifiable aetiology, as suggested by the AHA Scientific Statement on cardiomyopathies.^10^

CMR imaging played a central role in the diagnostic evaluation of our patient. The presence of diffuse myocardial oedema without late gadolinium enhancement suggested myocardial involvement without overt inflammatory injury or fibrosis.

SARS-CoV-2 infection has been associated with thyroid dysfunction, including unmasking of autoimmune thyroid disease. In our patient, however, positive thyroid autoantibodies and a previous history of hypothyroidism during pregnancy strongly support underlying Hashimoto’s thyroiditis, making a direct causal role of SARS-CoV-2 difficult to establish.

In our case, severe hypothyroidism was considered the most likely cause of myocardial and pericardial oedema. The contribution of the prior SARS-CoV-2 infection cannot be completely excluded; however, the mild troponin elevation, the absence of late gadolinium enhancement on CMR, and the rapid clinical response to thyroid hormone replacement therapy argue against SARS-CoV-2-related myopericarditis. Furthermore, normalization of thyroid function was accompanied by normalization of CPK and high-sensitivity cardiac troponin T levels and marked clinical improvement (*Table 1*), further supporting severe hypothyroidism as the most likely cause of the cardiac abnormalities.

## Conclusion

Severe hypothyroidism may present with myocardial and pericardial oedema mimicking inflammatory cardiac disease. Recognition of this reversible condition is essential, as appropriate thyroid hormone replacement therapy may lead to rapid clinical improvement. Thyroid function testing should therefore be considered in patients presenting with unexplained myocardial oedema or suspected myopericarditis.

## Data Availability

The data underlying this article are available within the article and its supplementary material.

## Lead author biography

Elena Gualini, Cardiologist at Niguarda Hospital, Milan, with a clinical focus on cardiomyopathies.

## Notes

**Consent:** Written informed consent was obtained from the patient for publication of this case report and any accompanying images in accordance with COPE guidelines.
